# Oestrogen inactivation in the colon: analysis of the expression and regulation of 17 **β** -hydroxysteroidehydrogenase isozymes in normal colon and colonic cancer

**DOI:** 10.1054/bjoc.2000.1324

**Published:** 2000-07-24

**Authors:** M A English, S V Hughes, K F Kane, M J S Langman, P M Stewart, M Hewison

**Affiliations:** Division of Medical Sciences, The Queen Elizabeth Hospital, The University of Birmingham, Birmingham, B15 2TH, UK

**Keywords:** colonic cancer, 17β-hydroxysteroid dehydrogenase, 1,25-dihydroxyvitamin D _3_, oestradiol, oestrone

## Abstract

Epidemiological data suggest that oestrogen contributes to the aetiology of colonic cancer. Furthermore, recent studies have suggested that local hormone metabolism may play a key role in determining colonic responsiveness to oestrogen. To further clarify this mechanism we have characterized the expression and regulation of isozymes of 17β-hydroxysteroid dehydrogenase (17β-HSD) in vitro and in situ. Immunohistochemistry was used to confirm expression of the type 2 and 4 isozymes of 17β-HSD (17β-HSD2 and 4) in normal colonic epithelial cells. Parallel studies suggested that both isozymes were abnormally expressed in colonic tumours and this was confirmed by Western blot analyses. Abnormal expression of 17β-HSD2 and 4 proteins was also observed in Caco-2, HT-29 and SW620 colonic cancer cell lines, although the overall pattern of oestrogen metabolism in these cells was similar to that seen in primary colonic mucosal tissue. The predominant activity (conversion of oestradiol to oestrone) was highest in Caco-2>SW620>HT-29, which correlated inversely with the rate of proliferation of the cell lines. Regulatory studies using SW620 cells indicated that the most potent stimulator of oestradiol to oestrone inactivation was the antiproliferative agent 1,25-dihydroxyvitamin D _3_(1,25D _3_), whilst oestradiol itself inhibited 17β-HSD activity. Both oestradiol and 1,25D _3_ decreased mRNA for 17β-HSD2 and 4. Data indicate that the high capacity for inactivation of oestrogens in the colon is associated with the presence of 17β-HSD2 and 4 in epithelial cells. Abnormal expression of both isozymes in colonic cancer cells and the stimulation of oestrogen inactivation by the antiproliferative agent 1,25D _3_ highlights a possible role for 17β-HSD isozymes as modulators of colonic cell proliferation. © 2000 Cancer Research Campaign


*British Journal of Cancer* (2000) **83**(4), 550–558
© 2000 Cancer Research Campaign
doi: 10.1054/ bjoc.2000.1324, available online at http://www.idealibrary.com on IDEAL®

### Oestrogen inactivation in the colon: analysis of the expression and regulation of 17β-hydroxysteroid dehydrogenase isozymes in normal colon and colonic cancer

**MA English*, SV Hughes, KF Kane, MJS Langman, PM Stewart† and M Hewison**

Division of Medical Sciences, The Queen Elizabeth Hospital, The University of Birmingham, Birmingham B15 2TH, UK

**Summary** Epidemiological data suggest that oestrogen contributes to the aetiology of colonic cancer. Furthermore, recent studies have suggested that local hormone metabolism may play a key role in determining colonic responsiveness to oestrogen. To further clarify this mechanism we have characterized the expression and regulation of isozymes of 17β-hydroxysteroid dehydrogenase (17β-HSD) in vitro and in situ. Immunohistochemistry was used to confirm expression of the type 2 and 4 isozymes of 17β-HSD (17β-HSD2 and 4) in normal colonic epithelial cells. Parallel studies suggested that both isozymes were abnormally expressed in colonic tumours and this was confirmed by Western blot analyses. Abnormal expression of 17β-HSD2 and 4 proteins was also observed in Caco-2, HT-29 and SW620 colonic cancer cell lines, although the overall pattern of oestrogen metabolism in these cells was similar to that seen in primary colonic mucosal tissue. The predominant activity (conversion of oestradiol to oestrone) was highest in Caco-2>SW620>HT-29, which correlated inversely with the rate of proliferation of the cell lines. Regulatory studies using SW620 cells indicated that the most potent stimulator of oestradiol to oestrone inactivation was the antiproliferative agent 1,25-dihydroxyvitamin $D_3$ (1,25$D_3$), whilst oestradiol itself inhibited 17β-HSD activity. Both oestradiol and 1,25$D_3$ decreased mRNA for 17β-HSD2 and 4. Data indicate that the high capacity for inactivation of oestrogens in the colon is associated with the presence of 17β-HSD2 and 4 in epithelial cells. Abnormal expression of both isozymes in colonic cancer cells and the stimulation of oestrogen inactivation by the antiproliferative agent 1,25$D_3$ highlights a possible role for 17β-HSD isozymes as modulators of colonic cell proliferation. © 2000 Cancer Research Campaign

**Keywords**: colonic cancer; 17β-hydroxysteroid dehydrogenase; 1,25-dihydroxyvitamin $D_3$; oestradiol; oestrone

Age and sex differences in the incidence of gastrointestinal cancers suggest the involvement of sex steroids. Specifically, post-menopausal loss of oestrogens in women appears to be associated with a lower risk of colonic cancer (Langman, 1967; Michael and Potter, 1982). These observations have been supported by studies in vitro which have highlighted the ability of active oestrogen (oestradiol, $E_2$) to stimulate the growth of colonic cancer cell lines (Xu and Thomas, 1994; Di Domenico et al, 1996). In spite of this, the precise mechanism by which oestrogens influence colonic cancer in vivo remains unclear, principally because of conflicting reports concerning the role and expression of receptors for $E_2$ (oestrogen receptors, ER) as determinants of oestrogen responsiveness in the colon (Francavilla et al, 1987; Jacobs et al, 1996). Di Domenico and colleagues suggested that the oestrogen-dependent growth of colonic cancer cells in vitro is dependent on ER expression (Di Domenico et al, 1996). However, although we have previously demonstrated differential responses to $E_2$ in pre-malignant and malignant cell lines (Singh et al, 1994), we were unable to correlate this with differences in ER expression (Singh et al, 1994, 1998). A further paradox is provided by epidemiological data which show that hormone replacement therapy (HRT) is associated with a lower risk of colonic cancer (Calle et al, 1995; Newcomb and Storer, 1995; Persson et al, 1996), although this may reflect differences in the composition and route of administration of HRT regimes.

To clarify these observations we have carried out a series of investigations that have focused on the concept of 'pre-receptor regulation' as the principal determinant of oestrogen responsiveness in the colon. Analogous to well documented studies in breast cancer (O'Neill et al, 1988; Sasano et al, 1996), we have postulated that local steroid metabolism in the colon may play a key role in modulating the effects of oestrogens by determining the tissue availability of active $E_2$. In recent studies using tissue biopsies we have shown that the normal colonic mucosa has a high capacity for metabolism of $E_2$ (English et al, 1999). Furthermore, the predominant metabolic activity, inactivation of $E_2$ to oestrone ($E_1$), was significantly decreased in paired tumour biopsies. Conversion of $E_2$ to $E_1$ is catalysed by the enzyme 17β-hydroxysteroid dehydrogenase (17β-HSD) for which several isozymes have been identified (Peltoketo et al, 1999). The presence of 17β-HSD activity in the colon appears to be due to expression of the type 2 and 4 isozymes of 17β-HSD (17β-HSD2 and 4), and expression of mRNA for the latter was shown to be significantly decreased in tumours compared to normal mucosae. In this report we have used in vitro model systems and tissue analysis in situ to examine further the relationship between 17β-HSD expression, colonic cell

*Received 14 January 2000*
*Revised 10 March 2000*
*Accepted 19 April 2000*

*Correspondence to:* M Hewison

*Recipient of a British Digestive Foundation Hunt Memorial/Hurst Centenary Grant

†Medical Research Council Senior Clinical Fellow proliferation and tumour development. Data provide further evidence for the importance of 17β-HSD2 and 4 as attenuators of $E_2$ bioavailability in the colon, and emphasize a possible role for 17β-HSD2 and 4 in the pathogenesis of colon cancer.

### MATERIALS AND METHODS

### Immunohistochemical studies

Colonic tumour and paired normal mucosal tissue were obtained with agreement from the local ethical approval committee. Five-micron thick, formalin-fixed tissue sections were cut and placed on coated glass slides. Sections were de-waxed and endogenous peroxidase activity quenched with 3% hydrogen peroxide. Sections were then incubated in donkey serum (Binding Site, Birmingham, UK) diluted 1/10 in PBS (15 min), followed by primary antibody diluted in PBS (1 hour). Antisera used were as follows: 17β-HSD2 monoclonal antiserum (1/500 dilution), a kind gift of Dr S Andersson (South Western Medical Center, Dallas, USA), was produced with a synthetic carboxyterminal peptide [C]RALRMP-NYKKKAT, corresponding to amino acids 375–387 in the human 17β-HSD2 protein; 17β-HSD4 monoclonal antibody (1:200 dilution), a kind gift of Dr J Adamski (GSF, Neuherberg, Germany) was prepared against the porcine 17β-HSD4 which cross-reacts with human, rat and mouse tissues. After washing, slides were incubated for 30 min with a biotinylated universal secondary antibody (Binding Site), diluted 1/100 in PBS, and binding visualised using ABC reagent (Binding Site) and 3,3′-diaminobenzidine (Sigma Chemical Co, Poole, UK). After staining, slides were washed and counterstained in Mayer's haematoxylin.

### Cell culture

Colonic carcinoma cell lines (SW620, Caco-2 and HT-29) were routinely maintained in Dulbecco's Modified Eagles Medium (DMEM), supplemented with 5% fetal calf serum (FCS) (both Life Technologies Ltd, Paisley, UK). Experimental cultures were carried out using phenol red-free DMEM supplemented with 5% charcoal-stripped FCS in the presence of absence of treatments (1–100 nM) which included: $E_1$, $E_2$, progesterone (Prg), dexamethasone (DEX), dihydrotestosterone (DHT), testosterone (T) (all Sigma) and 1α,25-dihydroxyvitamin $D_3$ (a kind gift from Dr M Uskokovic, Hoffman LaRoche, Nuttley, New Jersey).

### Measurement of 17β-HSD activity

Interconversion of $E_2$ to $E_1$ via 17β-HSD was assessed using previously reported methods (Hughes et al, 1997). Briefly, the substrates used were $^3$H-oestradiol ($^3$H-$E_2$) (specific activity: 110 Ci/mmol; Amersham, Little Chalfont, Buckinghamshire, UK) for measurement of oxidative 17β-HSD activity ($E_2$ to $E_1$), and $^3$H-oestrone ($^3$H-$E_1$) (specific activity; 80 Ci/mmol; Amersham) for reductive activity ($E_1$ to $E_2$). Assays were carried out in triplicate using substrate concentrations of between 25 nM and 2 μM. Medium was changed to serum-free DMEM 2 hours prior to enzyme assay and cells were then incubated in a further aliquot of serum-free medium containing $^3$H-$E_2$ or $^3$H-$E_1$. Reaction mixtures were extracted in chloroform and then separated on silica thin layer chromatography (TLC) plates in chloroform:ethyl-acetate (80:20 v/v). Conversion of tritiated steroid was measured using a Bioscan System 200 imaging TLC plate scanner (Bioscan Inc, Washington DC, USA), and the fractional conversion of $E_2$ to $E_1$, or $E_2$ to $E_1$ calculated. Residual cell monolayers were lysed and proteins analysed using standard Biorad protein assay (Biorad, Hemel Hempstead, UK). Activity was expressed as pmol product $h^{-1}$ mg protein$^{-1}$.

### Analysis of cell proliferation

Colonic cells were incubated with 0.5 μCi $^3$H-thymidine (specific activity 80 Ci mmol$^{-1}$; Amersham) for the last 6 hours of culture incubation. Unlabelled thymidine was added for the last 5 minutes to displace any non-specific uptake of $^3$H-thymidine. Cells were then washed in PBS and cellular proteins precipitated with cold 5% trichloroacetic acid. After removing the liquid layer, an aliquot of 0.1 M sodium hydroxide was added to the cells, and radioactivity in the resulting solubilized nuclear material was determined by scintillation counting. Data were reported as mean ± standard deviation of radioactive counts per minute (cpm) ($n = 4$).

### Analysis of 17β-HSD isozyme mRNA expression

#### *RNA extraction and RT-PCR analysis*

Total RNA was extracted from cultured cells using RNazol (AMS Biotechnology, Witney, UK), according to an adapted guanidinium-isothiocyanate method (Hughes et al 1997). Reverse transcription of RNA was performed using a Promega Reverse Transcription System (Promega Corp., Madison, WI) using previously reported methods (Hughes et al 1997). PCR analysis of 17β-HSD mRNA expression was carried out using the following primers for 17β-HSD types 1 to 4: 17β-HSD1: (5′ primer) 5′ AGG CTT ATG CGA GAG TCT GG3′; (3′ primer) 5′ CAT GGC GGT GAC GTA GTT GG3′ (bp 1460–1809); 17β-HSD2: 5′ CTG AGG AAT TGC GAA GAA CC3′, 5′ GAA GTC CTT GCT GGC TAA CG3′ (bp 445–1038); 17β-HSD3: 5′ ACA ATG TCG GAA TGC3′, 5′ AGG TTG AAG TGC TGG TCT TCT GC3′ (bp 437–1051); 17β-HSD4: 5′ CTA TTG GCC AGA AACTCC CT3′; 5′ GGA CCT TGG TTT GAA AAT GA3′ (bp 1028–1819). PCR reactions were set up in PCR buffer containing 50 mM KCl, 10 mM Tris-HCl (pH 9.0) and 0.1% Triton X-100, 1.5 mM $MgCl_2$, 0.2 μM of each dNTP, 0.5 μM (17β-HSD1, 2 and 3) or 0.4 μM (17β-HSD4) of primers and 1 μ of Taq DNA polymerase. Amplification of cDNA was performed using an initial denaturation step of 95°C followed by either 30 cycles of 95°C (1 min); 60°C (1 min); 72°C (1 min)(17β-HSD1–3) or 30 cycles of 95°C (1 min); 55°C (1 min); 72°C (1 min)(17β-HSD4). A final elongation step of 72°C for 7 minutes was also included.

#### *Northern blot analysis of 17β-HSD expression*

Northern blot analysis of 17β-HSD mRNA expression was carried out using aliquots (10 μg) of total RNA from each cell line. RNA was separated by denaturing gel electrophoresis and blotted onto Hybond N nylon filters (Amersham). After fixation by UV irradiation, filters were probed using previously reported methods (Hughes et al, 1997), and then exposed to Dupont Cronex film (Dupont/NEN, Boston) for various time periods, before development of autoradiographs.

#### *Western blot analysis*

Colonic mucosae, tumour tissue and cell lines were homogenized in the presence of the protease inhibitor PMSF (Sigma) (0.5 mM)

© 2000 Cancer Research Campaign and then centrifuged at 4°C and 6500 rpm for 5 min. Aliquots of the resulting supernatants, corresponding to cytoplasmic preparations, were then denatured at 95°C in 2% SDS, 10% glycerol, 62.5 mM Tris (pH 6.8) and size-separated on 10% SDS-PAGE gels. Proteins were transferred to Immobilon P membrane (0.4 μm; Millipore Corp, Bedford, MA). The resulting membranes were blocked by incubating overnight in PBS containing 10% bovine serum albumin (Sigma). Immunoreactivity was detected by incubation with primary antibody (17β-HSD2 diluted 1:100; 17β-HSD4 diluted 1:200) followed by peroxidase-conjugated anti-mouse secondary antibody (Amersham). The reaction detected by enhanced chemiluminescence (ECL, Amersham Pharmacia Biotech, Buckinghamshire, UK). The sizes of the reactive immunoproteins were estimated by comparison to the mobility of protein standards (Amersham).

### Data analysis

Immunohistochemistry and Western blot analyses were carried out using paired normal and tumour samples ($n$ = 3). Immunohistochemistry data were shown as a single pair of representative sections. Assays for 17β-HSD activity were carried out in triplicate and cell proliferation assays in quadruplicate. Data from both were reported as mean ± standard deviation (SD). Figures show typical experiments that were successfully repeated at least three times. Statistical calculations were performed using one-way analysis of variance (ANOVA) with the statistical software package Sigma-Stat3 (Jandel Scientific, Germany).

### RESULTS

### Expression of 17β-HSD2 and 4 in the colon

Immunocytochemical analysis of proteins for 17β-HSD2 and 4 in human tissue was carried out using colon sections as well as positive and negative control tissues (Figure 1). Analysis of positive control tissue (placenta) showed expression of 17β-HSD2 in stromal cells but not syncytiotrophoblasts, while 17β-HSD4 was detectable in both stromal and trophoblastic cells (Figure 1A and 1D). No staining was detected for either isozyme in testis (data not shown). In the colon, 17β-HSD2 and 4 were found mainly in colonic epithelial cells with increased immunoreactivity towards the luminal surface (Figure 1D and 1E). In contrast to normal colonic epithelium, expression of 17β-HSD2 and 4 was relatively weak in colonic mucosae adjacent to a tumour, and weaker still in tumour tissue itself (Figure 1C and 1F).

Western blot analysis of 17β-HSD2 and 4 indicated that the pattern of protein expression for these isozymes was different in tumour biopsies when compared to paired normal mucosal tissue (Figure 2). In normal mucosae and positive control tissue (placenta), 17β-HSD2 was expressed as a single protein species of 45 kDa. However, in each of the paired tumour samples an additional band of approximately 50 kDa was also detected. Similar data were obtained following Western analysis of 17β-HSD4 (Figure 3). In control tissue (placenta) and normal colonic mucosae a single protein species of 32 kDa was detected, with an additional band of approximately 46 kDa in tumour specimens. Statistical analysis showed no significant difference in expression between normal and tumour tissue for both 17β-HSD2 and 17β-HSD4 ($P$ = 0.386 and 0.318 respectively).

### Analysis of 17β-HSD activity in vitro

Further analysis of the expression and regulation of 17β-HSD2 and 4 in colonic epithelial cells was carried out using three colonic cancer cell lines. Initial enzyme activity studies revealed a similar pattern of oestrogen metabolism to that previously described in primary colonic mucosae (English et al, 1999). In Caco-2, SW620 and HT-29 cells the predominant 17β-HSD activity was oxidative conversion of $E_2$ to $E_1$. Using 50 nM $E_2$ as substrate the highest activity was observed in Caco-2 cells (52 ± 5.0 pmoles $h^{-1}$ mg protein$^{-1}$, followed by SW620 cells (23 ± 2.0 pmoles $h^{-1}$ mg protein$^{-1}$), and HT-29 cells (8 ± 1 pmoles $h^{-1}$ mg protein$^{-1}$). Kinetic analysis of 17β-HSD activity in SW620 cells indicated that the affinity constant ($K_m$) was 400 nM $E_2$, with a maximal enzyme activity ($V_{max}$) of 190 pmoles $E_1$ $h^{-1}$ mg protein$^{-1}$. Further enzyme assays using lysates from SW620 cells demonstrated that the conversion of $E_2$ to $E_1$ in these cells was dependent upon NAD+ as a co-factor (data not shown). Reductive activity (conversion of $E_1$ to $E_2$) was highest in Caco-2 cells (8.4 ± 3.8 pmoles $hr^{-1}$ mg protein$^{-1}$), followed by HT-29 (4.1 ± 2.3 pmoles $hr^{-1}$ mg protein$^{-1}$) and SW620 (2.5 ± 1.1 pmoles $hr^{-1}$ mg protein$^{-1}$).

All three cell lines were used in further studies to investigate the regulation of $E_2$ inactivation in colonic cells. Cells were incubated for 24 hours in charcoal-stripped, phenol red-free medium in the presence of various steroid hormones (all at 100 nM). Data shown in Figure 3 indicated that, following 24 h treatments, only 1,25$D_3$ and $E_2$ itself were able to modulate 17β-HSD activity. In Caco-2 and SW620, $E_2$ significantly inhibited oxidative 17β-HSD activity. In contrast, in SW620 and HT-29 cells, 1,25$D_3$ potently stimulated 17β-HSD activity. Caco-2 cells showed a similar trend which was not statistically significant at this time point. Further studies were carried out to investigate these responses in more detail and compare changes in 17β-HSD activity with effects on colonic cell proliferation. Dose–response experiments using SW620 cells confirmed the sensitive up-regulation of oxidative 17β-HSD activity by 1,25$D_3$, as well as the inhibitory effects of $E_2$ (Figure 4). However, it was noted that low doses (1 nM) of $E_1$ and testosterone (T) also produced a significant decrease in 17β-HSD activity. Although these effects were observed after 24 hours, we were unable to detect any significant changes in cell proliferation until 72 hours of treatment; at this time point antiproliferative effects were observed following treatment with 1,25$D_3$ or $E_1$ (Figure 4). Similar observations were also made using Caco-2 cells, and in both cell lines none of the treatments had a significant effect on reductive 17β-HSD activity ($E_1$ to $E_2$ conversion) (data not shown).

### 17β-HSD isozyme expression in colonic cancer cells

RT-PCR analyses indicated that transcripts for 17β-HSD1–4 were detectable in all three cell lines (Figure 5A). However, Northern blots probed with purified cDNAs generated by RT-PCR confirmed the presence of mRNA for 17β-HSD2 and 4, but not 17β-HSD1 and 3. Data in Figure 5B show Northern blot analyses of 17β-HSD2 and 4 mRNA expression in SW620 cells. Single transcripts corresponding to the reported mRNA species for 17β-HSD2 (1.3 kb) and 17β-HSD4 (3.0 kb) were detected. The expression of mRNA for both isozymes was inhibited by 24 hour treatment with 1,25$D_3$, $E_1$ and $E_2$ but not progesterone (all at 100 nM). Further analysis of the effects of 1,25$D_3$ indicated that lower doses of the hormone (1 nM and 10 nM) also down-regulated mRNA

© 2000 Cancer Research Campaign

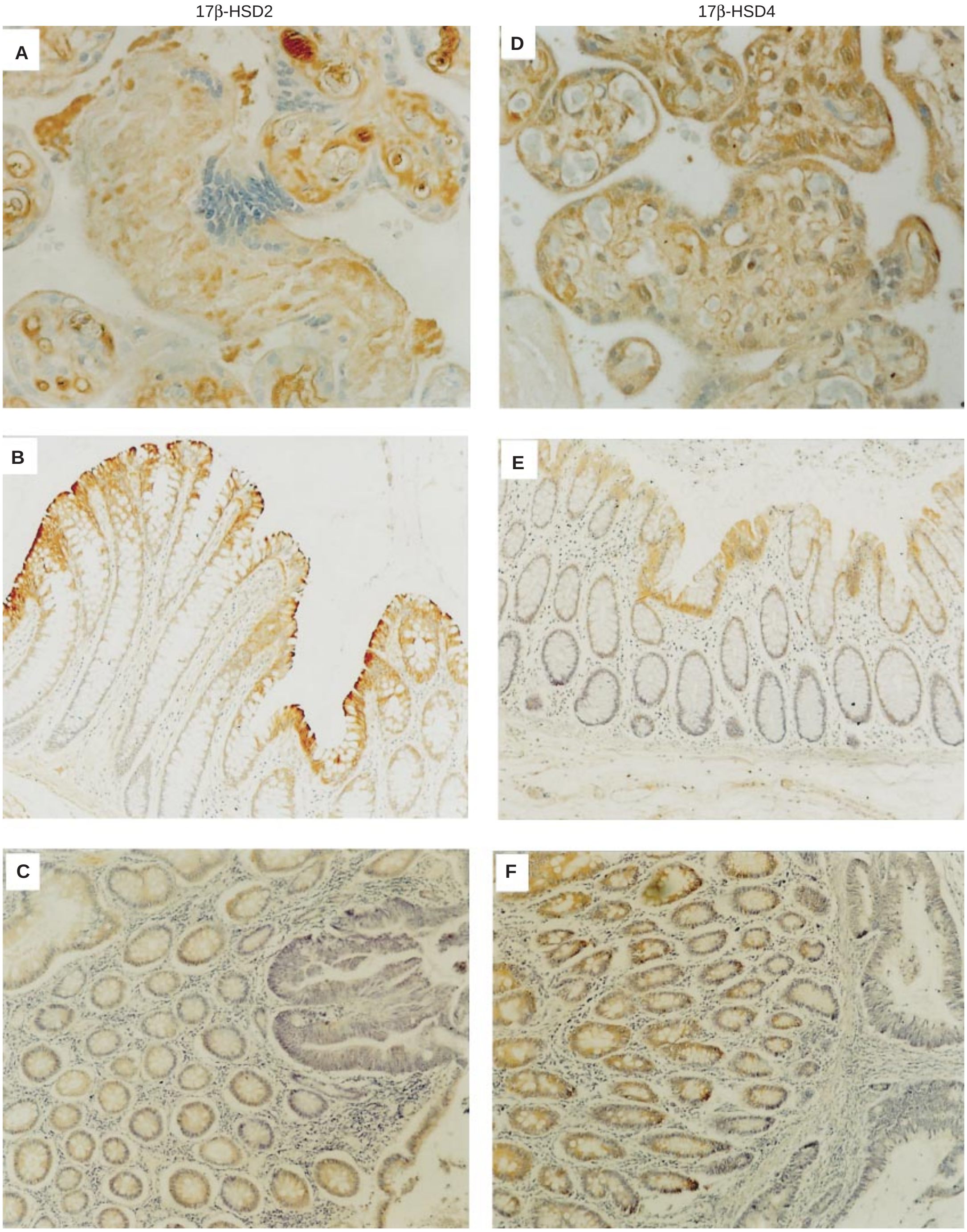

**Figure 1** Immunolocalization of 17β-HSD2 (**A**–**C**) and 17β-HSD4 (**D**–**F**) in: (**A** and **D**) placenta (positive control) (× 100); (**B** and **E**) colon surface epithelial cell layer (× 100); (**C** and **F**) colonic mucosa adjacent to a tumour (left) and colonic tumour tissue (right) (× 100). Positive staining (brown) is observed in colonic epithelial cells. Sections were counterstained with Mayer's haematoxylin levels for 17β-HSD2 and 4 (data not shown). The effects of $1,25D_3$ on expression of 17β-HSD2 and 4 were studied further using Western blots and isozyme-specific antibodies. Data in Figure 6 show that proteins corresponding to both isozymes were readily detectable in SW620 cells. In untreated cells two principal species were detected for 17β-HSD2 and 4. In each case the smallest protein band (45 kDa for 17β-HSD2, 32 kDa for 17β-HSD4) corresponded to the reported size of each isozyme. However, a larger species (approximately 50 kDa for 17β-HSD2, 46 kDa for 17β-HSD4) was also observed which was not present in positive control tissue (placenta). Treatment with $1,25D_3$ (1–100 nM) for 24 h did not appear to have any effect on the expression of the protein species for either 17β-HSD2 or 4.

### DISCUSSION

Sex hormones play a key role in the pathophysiology of some cancers. In particular, oestrogen has been shown to play a major

© 2000 Cancer Research Campaign

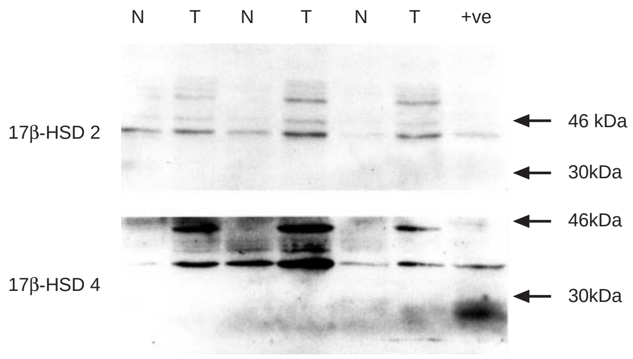

**Figure 2** Representative Western blot analysis of 17β-HSD2 and 4 expression in $n = 3$ paired samples of normal human colonic mucosae and colonic tumours. The position of molecular weight markers is shown on the right

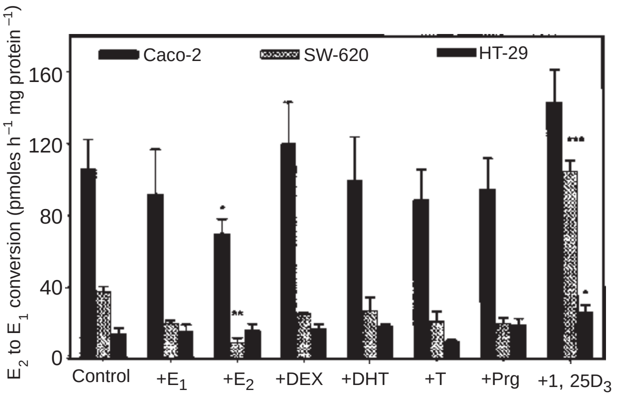

**Figure 3** Effect of steroid hormones on 17β-HSD activity in colonic cancer cells. Caco-2, SW-620 and HT29 cells were grown in phenol red-free medium +5% FCS until 80% confluent, and then for a further 24 hours in phenol red-free medium +5% charcoal-stripped FCS in the presence of: oestrone ($E_1$), oestradiol ($E_2$), dexamethasone (DEX), dihydrotestosterone (DHT), testosterone (T), progesterone (Prg), and 1,25-dihydroxyvitamin $D_3$ ($1{,}25D_3$) (all at 100 nM). Oxidative 17β-HSD activity was assessed using 100 nM [$^3$H]-$E_2$ as substrate. Data shown are means ± SD ($n = 3$). * = significantly different from control cells, $P < 0.05$;** = significantly different from control cells, $P < 0.01$; *** = significantly different from control cells, $P < 0.001$

role in the development, progression and treatment of breast cancer (Colditz, 1998; Osborne, 1998). This has led to the use of synthetic receptor agonists such as tamoxifen as a treatment regime for ER-positive breast tumours (McDonnell, 1999). However, another approach to this type of therapy has been to regulate ER responses by modulating the availability of endogenous ER ligand within tumour tissue. Local synthesis of oestrogens as a result of endogeneous aromatase, 17β-HSD and oestrone sulphatase activity has been demonstrated in endometrial (Maentausta et al, 1992), prostate (Elo et al, 1996) and breast tumours (Pasqualini et al, 1996). As a consequence, novel anti-cancer therapies have been aimed at controlling the local build-up of oestrogens in tumours by inhibiting the activity of specific steroidogenic enzymes, particularly aromatase and oestrone sulphatase (Brodie et al, 1999). Epidemiological evidence suggests that the incidence of colonic cancer is also influenced by sex hormones (Langman, 1967; Michael and Potter, 1982; Calle et al, 1995; Newcomb and Storer, 1995; Persson et al, 1996). In common with breast cancer, normal and neoplastic colonic mucosae show differential responses to $E_2$, although this does not appear to be due to dysregulation of ER expression (Singh et al, 1994, 1998).

In more recent studies we have postulated that enhanced oestrogen responsiveness in colonic tumours may be due to decreased inactivation of $E_2$ by isozymes of 17β-HSD (English et al, 1999). Immunohistochemical data presented here indicate that 17β-HSD2 and 4 are localized predominantly within the luminal surface of the normal colonic epithelium, supporting previous reports of 17β-HSD2 expression in the gastrointestinal epithelium of the mouse (Mustonen et al, 1998), and human fetus (Takeyama et al, 2000). It would therefore appear that oestrogen inactivation by locally expressed 17β-HSD isozymes is a feature of normal gastrointestinal biology. The most likely function of these isozymes within colonic epithelial cells is that they form part of a mechanism that protects the colon against environmental or bacterially-synthesized steroids. In recent studies, 17β-HSD2 has been shown to metabolize several orally administered steroidal compounds, including those used in oral contraceptives and HRT (Puranen et al, 1999). It was also interesting to note the presence of 17β-HSD2 and 4 in colonic crypts. Pluripotent stem cells occur in the crypt base and daughter cells migrate upwards undergoing a series of divisions before full maturation. In view of the mitogenic nature of $E_2$ this suggests that 17β-HSD2 and 4 may also play a role in modulating epithelial cell development.

Western blot analysis of normal colon of biopsy specimens confirmed the specificity of the 17β-HSD antisera used for immunohistochemistry studies. However, in tumour material and colonic cancer cell lines, additional larger protein species for 17β-HSD2 and 17β-HSD4 were also detected. Previous studies of 17β-HSD expression in breast cancer tissue, indicated the presence of enzymes with molecular weights in the range of 50–80 kDa in addition to a 35 kDa enzyme with different properties from those of the 35 kDa enzyme with the same molecular weight in adipose tissue (Mann et al, 1999). To date no similar studies have been carried out on colonic cancer tissue. However, it is possible to speculate that the presence of tumour-specific hydroxysteroid dehydrogenase proteins may alter co-factor availability, or result in the quenching of specific substrates. This may help to explain the decreased capacity for oestrogen inactivation that we have described previously in colonic tumours (English et al, 1999). It is also important to note that 17β-HSD4 is a 80 kDa multi-domain protein, which has previously been reported to undergo processing to the 32 kDa protein detected in target tissues. Evidence for further processing into additional fragments has been demonstrated in rat tissue by peroxisome proliferator chemicals (Fan et al, 1998). Tumour specific processing of this particular isozyme may therefore occur by an as yet unidentified mechanism, resulting in species that competitively alter oestrogen inactivation.

Experiments in vitro confirmed that the overall pattern of oestrogen metabolism in these cells was similar to that observed with primary human colonic tissue, namely 17β-HSD-mediated inactivation of $E_2$ to $E_1$. Levels of $E_2$ metabolism varied between the cell lines but only the antiproliferative agent $1{,}25D_3$ and $E_1$ and $E_2$ had any significant effect on enzyme activity. $E_2$-mediated regulation of oxidative 17β-HSD activity has not previously been reported in other tumour tissues although, in breast cancer cells, $E_1$ has been shown to significantly inhibit 17β-HSD activity (Mehta and Gupta, 1993; Peltoketo et al, 1996). The decreased 17β-HSD activity observed following treatment of Caco-2 and SW620 cells

*© 2000 Cancer Research Campaign*

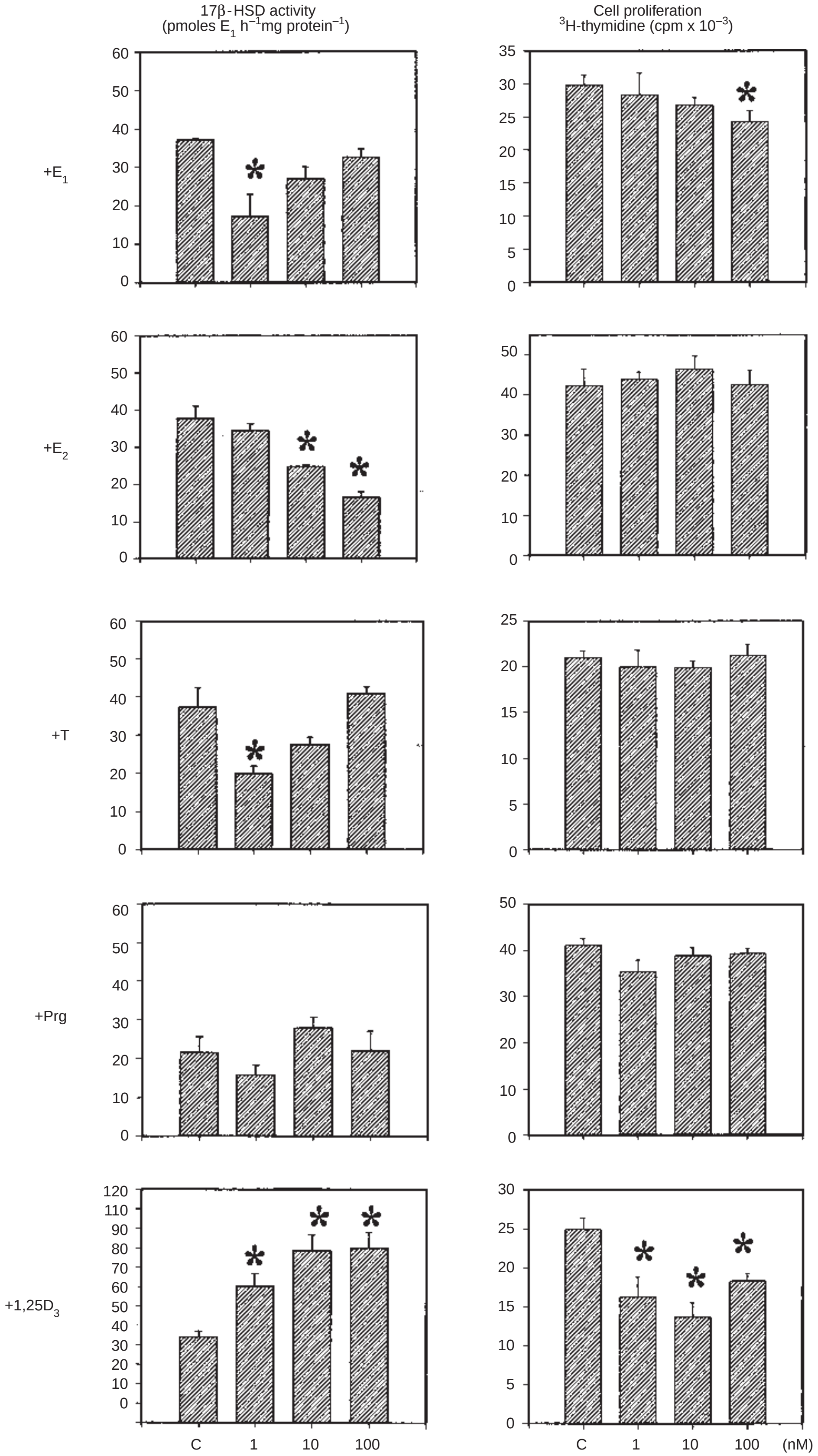

**Figure 4** Effects of steroid hormones on 17β-HSD activity and cell proliferation in SW-620 cells. Cell were treated with 1–100 nM concentrations of oestradiol ($E_2$), oestrone ($E_1$), testosterone (T), progesterone (Prg), or 1,25-dihydroxyvitamin $D_3$ (1,25$D_3$) for either 24 hours (17β-HSD activity) or 72 hours (cell proliferation). Data shown are means ± SD (*n* = 3 for 17β-HSD activity, *n* = 4 for $^3$H-thymidine). *= significantly different from control cells, $P < 0.05$; ** = significantly different from control cells, $P < 0.01$

*© 2000 Cancer Research Campaign* 

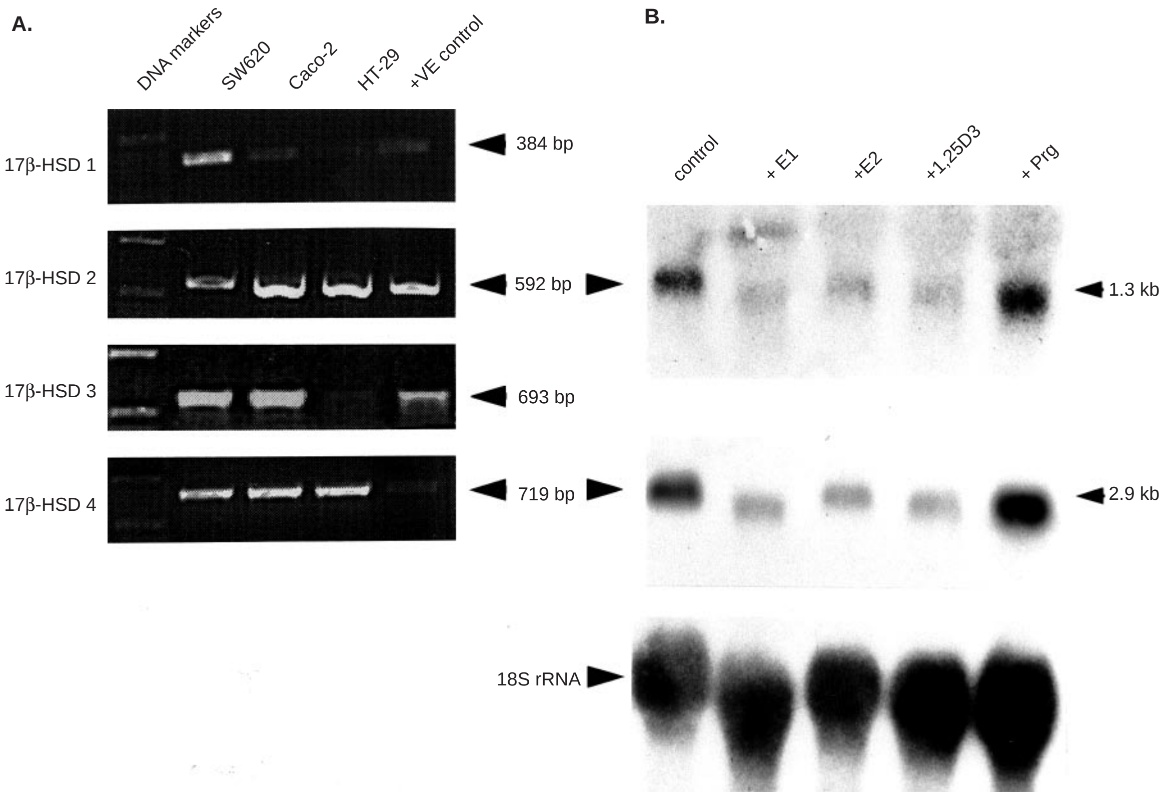

**Figure 5** Expression of mRNA for 17β-HSD isozymes in colonic cancer cells. (**A**) RT-PCR analysis of 17β-HSD isozyme expression in Caco-2, SW620 and HT29 cells. Positive control RNAs included placenta (17β-HSD1, 2 and 4) and testis (17β-HSD3). (**B**) Northern analysis of 17β-HSD2 and 4 expression in SW620 cells treated with oestradiol ($E_2$), oestrone ($E_1$), progesterone (Prg), or 1,25-dihydroxyvitamin $D_3$ (1,25$D_3$) for 24 hours (all at 100 nM). Blots were probed with radiolabelled PCR fragments generated from the studies shown in 4A

with $E_2$ and $E_1$ correlated with decreased expression of mRNAs for both 17β-HSD2 and 4. In contrast, although 1,25$D_3$ also down-regulated the expression of mRNAs for 17β-HSD2 and 4, its overall effect on enzyme activity in HT-29 and SW620 cells was to stimulate $E_2$ inactivation. It therefore seems likely that there are different mechanisms involved in regulating oestrogen metabolism in colonic cells. The effect of $E_1$ and $E_2$ appears to be due to direct inhibition of 17β-HSD transcription. In contrast, responses to 1,25$D_3$ were similar to those we have previously described for HL60 leukaemic cells and normal human keratinocytes; treatment with 1,25$D_3$ produced a similar rapid induction of $E_2$ inactivation that was also associated with decreased 17β-HSD4 mRNA expression (Hughes et al, 1997; Mountford et al, 1999). These observations, together with the Western analyses presented here suggest that the stimulation of oestrogen metabolism by 1,25$D_3$ in colonic cancer cells may not be mediated via direct regulation of 17β-HSD2 or 4. Rather it is possible that the 1,25$D_3$-induced stimulation of $E_2$ inactivation is due to indirect stimulation of another 17β-HSD isozyme, possibly as a result of a shift in the availability of enzyme co-factors such as NAD+. The contribution of other 17β-HSD isozymes to cell proliferation and differentiation remains unclear. The type 1, 3, 5 and 7 isozymes show predominantly reductive ($E_1$ and $E_2$) activity and are therefore unlikely candidates, particularly as we were unable to demonstrate significant amounts of mRNA for 17β-HSD1 and 3 in colonic mucosae and cancer cell lines. However, conversion of $E_1$ to $E_2$ was detectable in these samples. Although this was relatively low compared to oxidative activity ($E_2$ to $E_1$ conversion) the possibility remains that a variety of 17β-HSD enzyme activities are found in the colon, including possible novel isozymes of 17β-HSD.

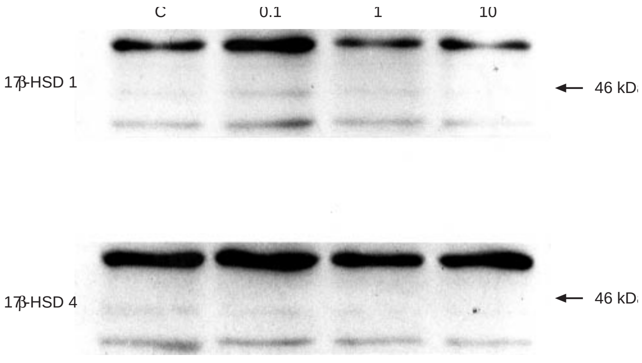

**Figure 6** Western blot analysis of 17β-HSD2 and 4 expression in SW-620 cells. Changes in the expression of proteins for 17β-HSD2 and 4 were assessed following treatment with oestradiol ($E_2$), oestrone ($E_1$), progesterone (Prg), or 1,25-dihydroxyvitamin $D_3$ (1,25$D_3$) for 24 hours (all at 100 nM). The position of molecular weight markers is indicated on the right

In previous reports we have postulated that decreased $E_2$ inactivation may make a significant contribution to the enhanced cell proliferation associated with colonic tumours (English et al, 1999). Here we have shown that the antiproliferative effects of 1,25$D_3$ are preceeded by sensitive up-regulation of $E_2$ inactivation. In contrast, known mitogenic agents such as $E_2$ demonstrated

© 2000 Cancer Research Campaign apparent autocrine inhibition of 17β-HSD activity. Similar inhibition of $E_2$ inactivation was also observed with low doses of $E_1$ or T, although the precise mechanism for this remains unclear. Other groups have demonstrated pro-proliferative responses to $E_2$ in colonic cells lines (Xu and Thomas, 1994; Di Domenico et al, 1996). Although we were unable to confirm this effect of $E_2$ in our models systems, it was interesting to note that treatment with $E_1$ at concentrations of 100 nM or greater significantly inhibited cell proliferation. These data support previous studies in which we have shown that generation of $E_1$ through the action of 17β-HSD isozymes may act as a novel component of cell differentiation processes (Hughes et al, 1997; Mountford et al, 1999). Thus, stimulation of 17β-HSD activity by established differentiating agents may not only decrease the availability of mitogenic $E_2$ but could also enhance local concentrations of antiproliferative $E_1$.

A putative role for $E_1$ as a novel antiproliferative agent may help to resolve the paradox associated with the epidemiology of oestrogens and colon cancer. On the one hand an increased ratio of circulating $E_1/E_2$ in postmenopausal women is associated with decreased risk of colon cancer. Conversely, HRT has also been shown to protect against colon cancer. A clear relationship between the composition of HRT and effects on colon cancer has yet to be fully described. However, it is important to recognize that the principal prescribed HRTs (Premarin/Prempak C) contain delta-8-$E_1$ sulphate rather than $E_2$. Consequently, within the colon, the most likely metabolite that will be derived from this treatment is $E_1$ and not $E_2$. Importantly, the immunohistochemistry data presented here indicate that orally administered HRT regimes that contain $E_2$ may lead to the generation of increased local levels of $E_1$ in the colon. The discrete expression of 17β-HSD2 and 4 in the epithelial cells of the colonic mucosa provides an efficient barrier for inactivation of ingested steroids including the $E_2$ present in some oral HRT regimes. Other components of HRT preparations such as progesterone appeared to be without effect in terms of 17β-HSD expression/activity, or cell proliferation. It is possible that progesterone itself may be subject to local metabolism but as yet the potential effects of this on colonic cells remain unclear.

In summary we have presented further evidence for the important role of 17β-HSD isozymes as modulators of oestrogen effects in the colon. Localization of 17β-HSD2 and 4 to the epithelial layer of the colon and the presence of these isozymes in epithelial cell lines highlights a potential role as protective barrier against ingested oestrogens. In addition, regulatory studies have confirmed the association between colonic cell proliferation and 17β-HSD activity, further emphasizing the possible importance of $E_2$ inactivation in the aetiology of colon cancer. Further studies to define the cellular impact of HRT, and to identify other 17β-HSD isozymes in the colon will help to clarify the role of hormone metabolism in colonic cell development, but may also provide a novel target for improved therapies for colonic cancer.

### ACKNOWLEDGEMENTS

The authors are grateful to Dr Daniel Zehnder and Mrs Marie C Williams (Birmingham) for their help in preparing colonic tissue sections, and Professors Adamski (GSF, Neuherberg, Germany) and Andersson (Southwestern Medical Center, Dallas, USA) for kindly providing antisera. We would also like to thank Dr WE Rainey (SouthWestern Medical Center, Dallas) for advice regarding analysis of 17β-HSD expression.

### REFERENCES

Brodie A, Lu Q and Long B (1999) Aromatase and its inhibitors. *J Steroid Biochem Mol Biol* **69**: 205–210

Calle EE, Miracle-McMahill HL, Thun MJ and Heath Jr CW (1995) Estrogen replacement therapy and risk of fatal colon cancer in a prospective cohort of postmenopausal women. *J Natl Cancer Inst* **87**: 517–523

Colditz GA (1998) Relationship between estrogen levels, use of hormone replacement therapy, and breast cancer. *J Natl Cancer Inst* **90**: 814–823

Di Domenico M, Castoria G, Bilancio A, Migliaccio A and Auricchio F (1996) Estradiol activation of human colon carcinoma-derived Caco-2 cell growth. *Cancer Res* **56**: 4516–4521

Elo JP, Akinola LA, Poutanen M, Vihko P, Kyllonen AP, Lukkarinen O and Vihko R (1996) Characterization of 17beta-hydroxysteroid dehydrogenase isoenzyme expression in benign and malignant human prostate. *Int J Cancer* **66**: 37–41

English MA, Kane KF, Cruickshank N, Langman MJS, Stewart PM and Hewison M (1999) Loss of estrogen inactivation in colonic cancer. *J Clin Endocrinol Metab* **84**: 2080–2085

Fan L-Q, Cattley RC and Corton JC (1998) Tissue-specific induction of 17β-hydroxysteroid dehydrogenase type IV by peroxisome proliferator chemicals is dependent on the peroxisome proliferator-activator receptor α. *J Endocrinol* **158**: 237–246

Fomitcheva J, Baker ME, Anderson E, Lee GY and Aziz N (1998) Characterization of ke6, a new 17 beta-hydroxysteroid dehydrogenase, and its expression in gonadal tissues. *J Biol Chem* **273**: 22664–22671

Francavilla A, Dileo A, Polimeno L, Conte D, Barone M, Fanizza G, Chiumarulo C, Rizzo G and Rubino M (1987) Nuclear and cytosolic estrogen receptors in human colon carcinoma and in surrounding non-cancerous colonic tissue. *Gastroenterol* **93**: 1301–1306

Hughes SV, Robinson E, Bland R, Lewis HM, Stewart PM and Hewison M (1997) 1,25-Dihydroxyvitamin D3 regulates estrogen metabolism in cultured keratinocytes. *Endocrinol* **138**: 3711–3718

Jacobs E, Watson SA, Hardcstle JD and Robertson JFR (1996) Oestrogen and progesterone receptors in gastrointestinal cancer cell lines. *Eur J Cancer* **13**: 2348–2353

Langman MJS (1967) Current trends in the epidemiology of cancer of the colon and rectum. *Proc R Soc Med* **60**: 210–212

Maentausta O, Boman K, Isomaa V, Stendahl U, Backstrom T and Vihko R (1992) Immunhistochemical study of the human 17 beta-hydroxysteroid dehydrogenase and steroid receptors in endometrial adenocarcinoma. *Cancer* **70**: 1551–1555

Mann VZ, Newton CJ and Tait GH (1991) 17β-hydroxysteroid dehydrogenases in human breast tissues: purification and characterization of soluble enzymes and the distribution of particulate and soluble forms in adipose, non-adipose and tumour tissues. *J Mol Endocrinol* **7**: 45–55

McDonnell DP (1999) The molecular pharmacology of SERMs. *Trends Endocrinol Metab* **10**: 301–311

McMichael AJ and Potter JD (1982) Colon cancer and sex hormones. *Lancet* **1**: 1190–1192

Mehta RR and Das Gupta TK (1993) Regulation of 17 beta-hydroxysteroid dehydrogenase in a newly-established human breast carcinoma cell line. *J Steroid Biochem Mol Biol* **46**: 623–629

Mountford JC, Bunce CM, Hughes SV, Drayson MT, Webb D, Brown G and Hewison M (1999) Estrone potentiates myeloid cell differentiation: A role for 17β-hydroxysteroid dehdrogenase in modulating haemopoiesis. *Exp Hematol* **27**: 451–460

Mustonen MVJ, Poutanen MH, Kellokumpu S, de Launoit Y, Isomaa VV, Vihko RK and Vihko PT (1998) Mouse 17 beta-hydroxysteroid dehydrogenase type 2 mRNA is predominantly expressed in hepatocytes and in surface epithelial cells of the gastrointestinal and urinary tracts. *J Mol Endocrinol Metab* **78**: 197–204

Newcomb PA and Storer BE (1995) Postmenopausal hormone use and risk of large bowel cancer. *J Natl Cancer Inst* **87**: 1067–1071

O'Neill JS, Elton RA and Miller WR (1988) Aromatase activity in adipose-tissue from breast quadrants – a link with tumour site. *BMJ* **296**: 741–743

Osborne CK (1998) Steroid hormone receptors in breast cancer management. *Breast Cancer Res Treat* **51**: 227–238

Pasqualini JR, Chetrie G, Blacker C, Feinstein MC, Delelonde L, Talbi M and Maloche C (1996) Concentrations of estrone, estradiol and estrone sulfate and evaluation of sulfatase and aromatase activities in pre- and postmenopausal breast cancer patients. *J Clin Endo Metab* **81**: 1460–1464

Peltoketo H, Isomaa V, Poutanen M and Vihko R (1996) Expression and regulation of 17β-hydroxysteroid dehydrogenase type 1. *J Endocrinol* **150**: (Suppl): S21–S30

© 2000 Cancer Research Campaign 

Peltoketo H, Vihko P and Vihko R (1999) Regulation of estrogen action: Role of 17 beta-hydroxysteroid dehydrogenase. *Vitamins and Hormones* **55**: 353–398

Persson I, Yuen J, Bergvist L and Schairer C (1996) Cancer incidence and mortality in women receiving estrogen and estrogen-progestin replacement therapy – long term follow-up of Swedish cohort. *Int J Cancer* **67**: 327–332

Puranen TJ, Kurkela RM, Lakkakorpi JT, Poutanen MH, Itaranta PV, Melis JPJ, Ghosh D, Vihko RK and Vihko PT (1999) Characterization of molecular and catalytic properties of intact and truncated human 17β-hydroxysteroid dehydrogenase type 2 enzymes: intracellular localization of the wild-type enzyme in the endoplasmic reticulum. *Endocrinol* **140**: 3334–3341

Sasano H, Frost AR, Saitoh R, Harada N, Poutanen M, Vihko R, Bulun SE, Silverberg SG and Nagura H (1996) Aromatase and 17 beta-hydroxysteroid dehydrogenase type 1 in human breast carcinoma. *J Clin Endocrinol Metab* **81**: 4042–4046

Singh S, Paraskeva C, Gallimore PH, Sheppard MC and Langman MJS (1994) Differential growth response to oestrogen of premalignant and malignant colonic cell lines. *Anticancer Res* **14**: 1037–1042

Singh S, Poulsom R, Hanby AM, Rogers LA, Sheppard MC and Langman MJS (1998) Expression of oestrogen receptor and oestrogen-inducible genes pS2 and ERD5 in large bowel mucosa and cancer. *J Pathol* **184**: 153–160

Takeyama J, Suzuki T, Hirasawa G, Muramatsu Y, Nagura H, Iinuma K, Nakamura J, Kimura K, Yoshihama M, Harada N, Andersson S and Sasano H (2000) 17β-hydroxysteroid dehydrogenase type 1 and 2 expression in the human fetus. *J Clin Endo Metab* **85**: 410–416

Xu X and Thomas ML (1994) Estrogen receptor-mediated direct stimulation of colon cancer cell growth in vitro. *Mol Cell Endo* **105**: 197–201

 *© 2000 Cancer Research Campaign*
